# Real-time quality control of dripping pill’s weight based on laser detection technology

**DOI:** 10.1038/s41598-023-32805-z

**Published:** 2023-04-15

**Authors:** Xiaoping Wang, Hang Chen, Ying Tian, Haibin Qu

**Affiliations:** 1 Pharmaceutical Informatics Institute, College of Pharmaceutical Sciences, Zhejiang University, No. 866 Yuhangtang Road, Hangzhou, 310058 China; 2 State Key Laboratory of Component-Based Chinese Medicine, Innovation Center in Zhejiang University, Hangzhou, 310058 China

**Keywords:** Optical sensors, Characterization and analytical techniques, Pharmaceutics

## Abstract

The present work reports developing the first process analytical technology (PAT)-based real-time feedback control system for maintaining the *Ginkgo biloba* leaf dripping pills weight during manufacturing. The opening degree of the drop valve and the weight of dripping pills were chosen as the manipulated variable and as the controlled variable, respectively. A proportional-integral controller was programmed to automatically reach the desired dripping pills weight by adjusting the opening degree of the drop valve. The closed-loop feedback control system could automatically compensate for the disturbances and ensure a predefined weight of the dripping pills with excellent robustness, high accuracy, and high efficiency during manufacturing. Furthermore, the closed-loop feedback control system improved the process capability of the dripping process, and the process capability index was > 1.67. This study provides a new approach to real-time control of the weight of dripping pills and improves the process capability during *Ginkgo biloba* leaf dripping pills manufacturing.

## Introduction

*Ginkgo biloba* leaf extract is a typical herbal medicine that has been widely used in the clinic because of its specific therapeutic effects on cardiovascular and cerebrovascular diseases^1,2^. Many *Ginkgo biloba* leaf dosage forms have been used, such as tablets, oral solutions, injections, and dripping pills. *Ginkgo biloba* leaf dripping pills is an oral solid dosage form of solid dispersion extensively used in the clinic because of their excellent bioavailability and pharmacokinetics^3,4^. The weight of the dripping pills is an important quality attribute that determines the dose uniformity of active pharmaceutical ingredients^5,6^. The Pharmacopeia of the People’s Republic of China specified pill weight of *Ginkgo biloba* leaf dripping pills range from 52.8 to 67.2 mg^7^. The variation in the pill weight affects the dose uniformity and oral bioavailability. Therefore, improving the weight uniformity of dripping pills is one of the top priorities in pharmaceutical manufacturing.

In commercial production, the manufacturing of the dripping pills depends on the workers’ experience. The operators monitored and controlled the dripping pill weight by manipulating the valve of the drop head to adjust droplet size during the dripping process. This needs to weigh the droplets by an electric balance every 30 min. If the weight of the droplets was out of the acceptable range, the opening degree of the drop head valve was adjusted. The opening degree of the drop head valve affects the flow resistance of the dispersing liquid and further determines the weight of the droplet. However, this approach has several disadvantages, including low control accuracy, delay in quality control, experienced staff, and high labor intensity. Therefore, it is urgent to develop an active in-line control method to ensure the weight uniformity of dripping pills. Feedback control strategy provides an important approach to improve the reliability of pharmaceutical production processes in continuous manufacturing of pharmaceuticals^8–10^.

This work aimed to develop a real-time in-line feedback control system that uses laser detection technology. It can be used for real-time release testing of the weight of *Ginkgo biloba* leaf dripping pills. A self-designed software was programmed for building a real-time feedback control system with a proportional-integral (PI) controller and an in-line laser detection system. The opening degree of the head drop valve was chosen as the manipulated variable, and the measured weight of the dripping pills was used as the controlled variable. The functionality and robustness of the real-time feedback control system were investigated. The study results could enhance our understanding and propose a feasible real-time control strategy to improve the product quality during the dripping pills manufacturing process.

## Materials and methods

### Materials

The dripping experiment was conducted using a formulation containing *Ginkgo biloba* leaf extract (Zhejiang Conba Pharmaceutical Co., Ltd., Hangzhou, China) and polyethylene glycol 4000 (Wanbangde Pharmaceutical Group, Wenling, China). The dimethyl silicone oil was used as condensing oil (Jiangxi Alpha Hi-tech Pharmaceutical Co., Ltd., Pingxiang, China). The petroleum ether was used as condensing oil washing solvent (Sinopharm Chemical Reagent Co., Ltd., Shanghai, China). The authors confirm that the present study complies with the IUCN Policy Statement on Research Involving Species at Risk of Extinction and the Convention on the Trade in Endangered Species of Wild Fauna and Flora.

The weight of the dripping pills was in-line measured using a laser detection system which is equipped with a laser micrometer (KEYENCE IG-028, Shanghai, China), a sensor amplifier (KEYENCE IG-1000, Shanghai, China), and a data acquisition card (National Instrument cDAQ-9171, Shanghai, China). The weight of materials was weighed using an electric balance (Mettler Toledo AE240, Shanghai, China). The homogenous dispersing liquid was prepared using a circulating oil bath (Greatwall Scientific SY-20, Zhengzhou, China) and an electric mixer (Zhengrong instrument ES-60 M, Changzhou, China). The preparation of dripping pills was performed using a multifunction dripping machine (Anruikang, Beijing, China).

### Dripping experiments

**Figure 1:**
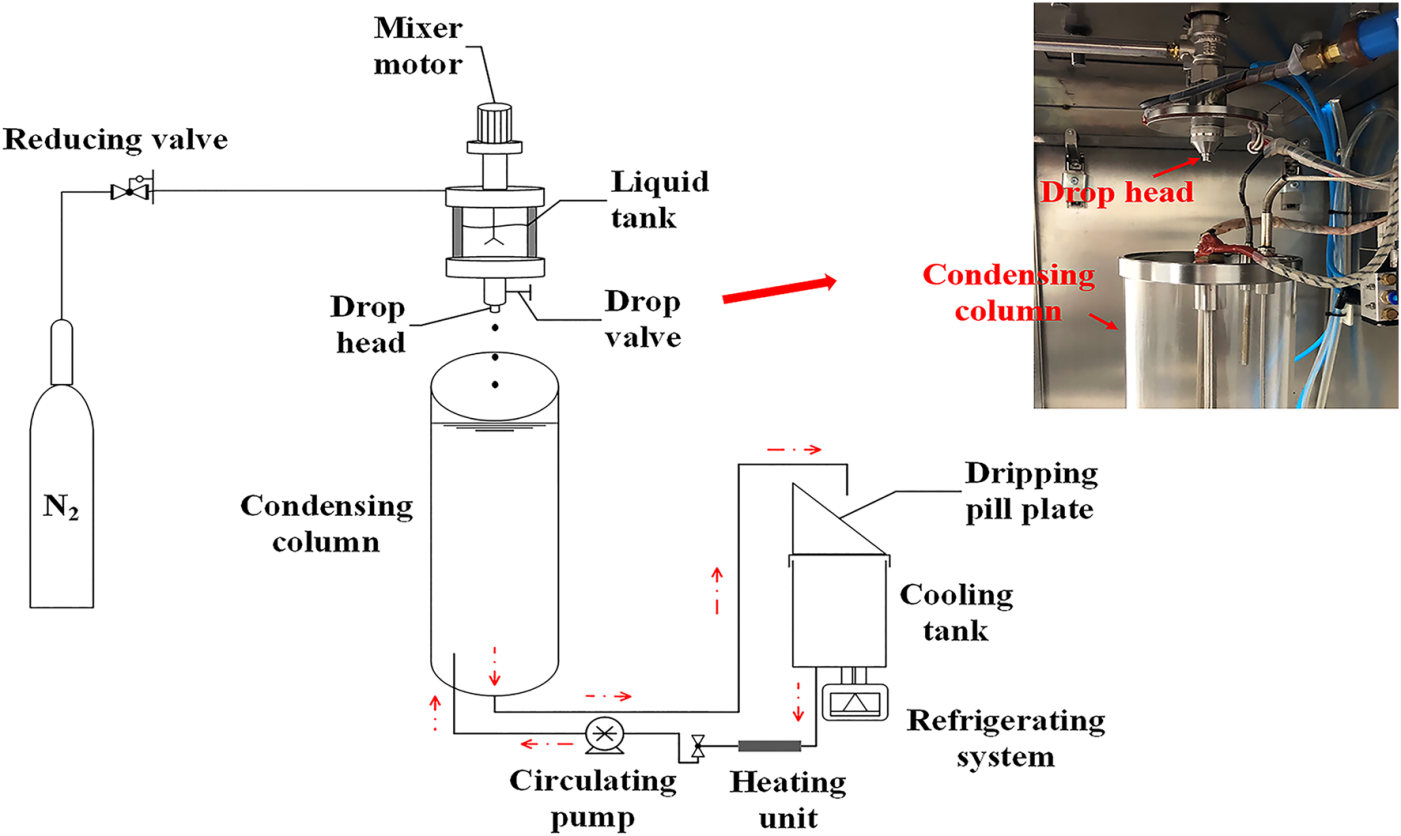
Schematic diagram of multifunctional dripping machine.

The dripping experiment was performed using a multifunction dripping machine (Fig. 1). The dripping machine consisted of a dripping unit and a condensing unit. The dripping unit was composed of a liquid tank, drop valve, drop head, and pressure device. The liquid tank was a double-layer tank in which the outer layer was equipped with a heating unit and filled with conduction oil. The condensing unit consisted of a condensing column and temperature control components. Dimethyl silicone oil was used as condensing oil and filled in the condensing column. A homogeneous dispersing liquid contained *Ginkgo biloba* leaf extract and polyethylene glycol 4000 (4:11, w/w) was performed and transferred to the liquid tank of the dripping machine. The temperature of the dispersing liquid in the liquid tank was kept at 80 °C. The hot dispersing liquid flowed out of the drop head and transferred to the condensing column. Then the spherical raw pills were formed under the force balance between the surface tension, gravity, and forming force. The drop distance was 75 mm. The temperature of the condensing oil was kept at 20 °C. Before the dripping process, the dispersing liquid and condensing oil temperatures were balanced for hours.

### Real-time dripping pills weight measurement by an in-line laser detection system

In this study, the weight of the dripping pills was used as a controlled variable. In our previous work, a laser detection system was constructed for in-line analysis and monitoring of the dripping process^11^. The functionality and robustness of the laser detection system have been investigated, and the results showed that the laser detection system has an excellent capability of pill weight quantification; hence we have chosen laser detection system as an in-line PAT tool to measure the weight of the dripping pills.

### Real-time feedback control

#### Identification of a suitable process parameter as a manipulated variable

**Figure 2:**
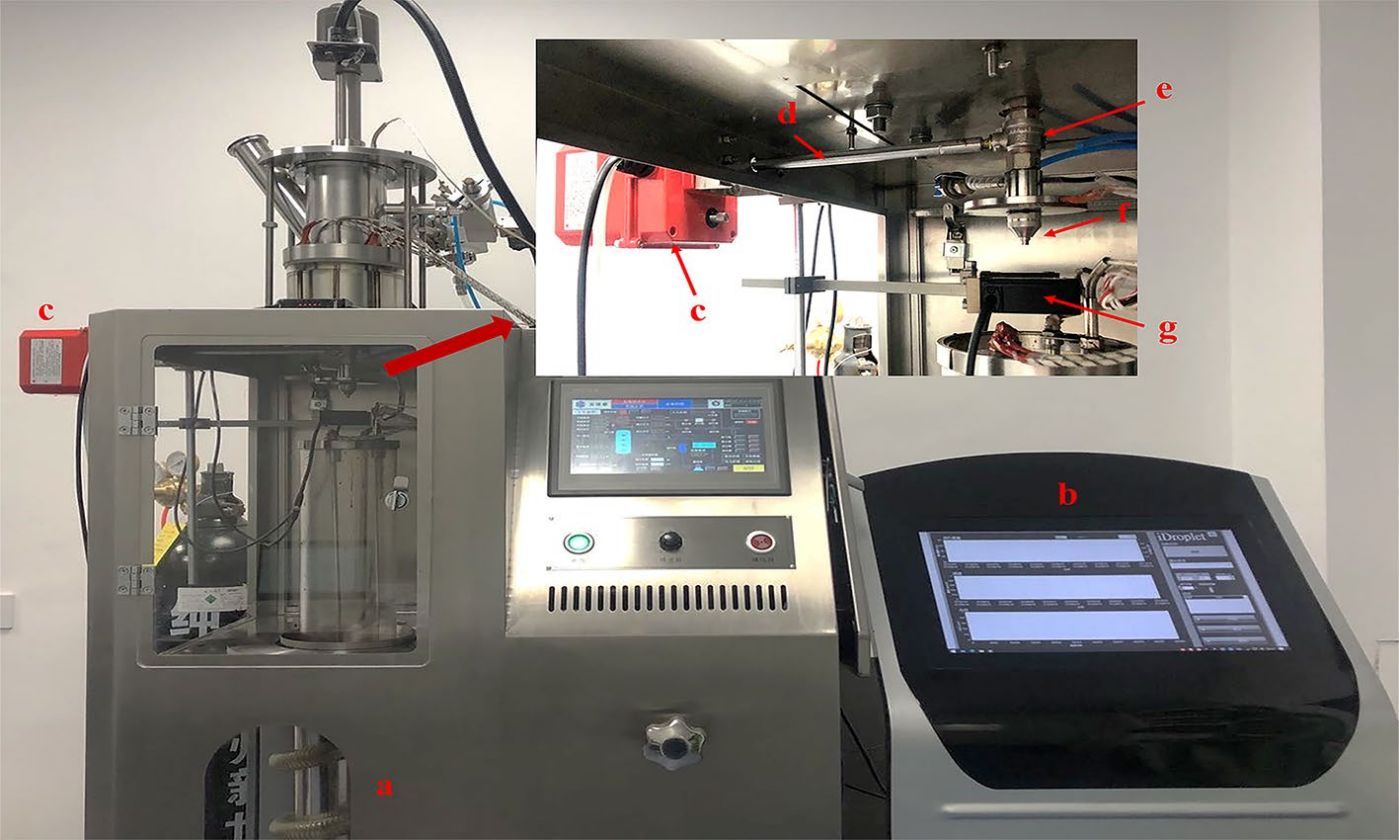
The setup used in the dripping experiments. (**a**) dripping machine, (**b**) self-designed software carrying out the data processing and controlling, (**c**) electric actuator, (**d**) valve rod, (**e**) drop valve, (**f**) drop head, and (**g**) laser micrometer.

**Figure 3:**
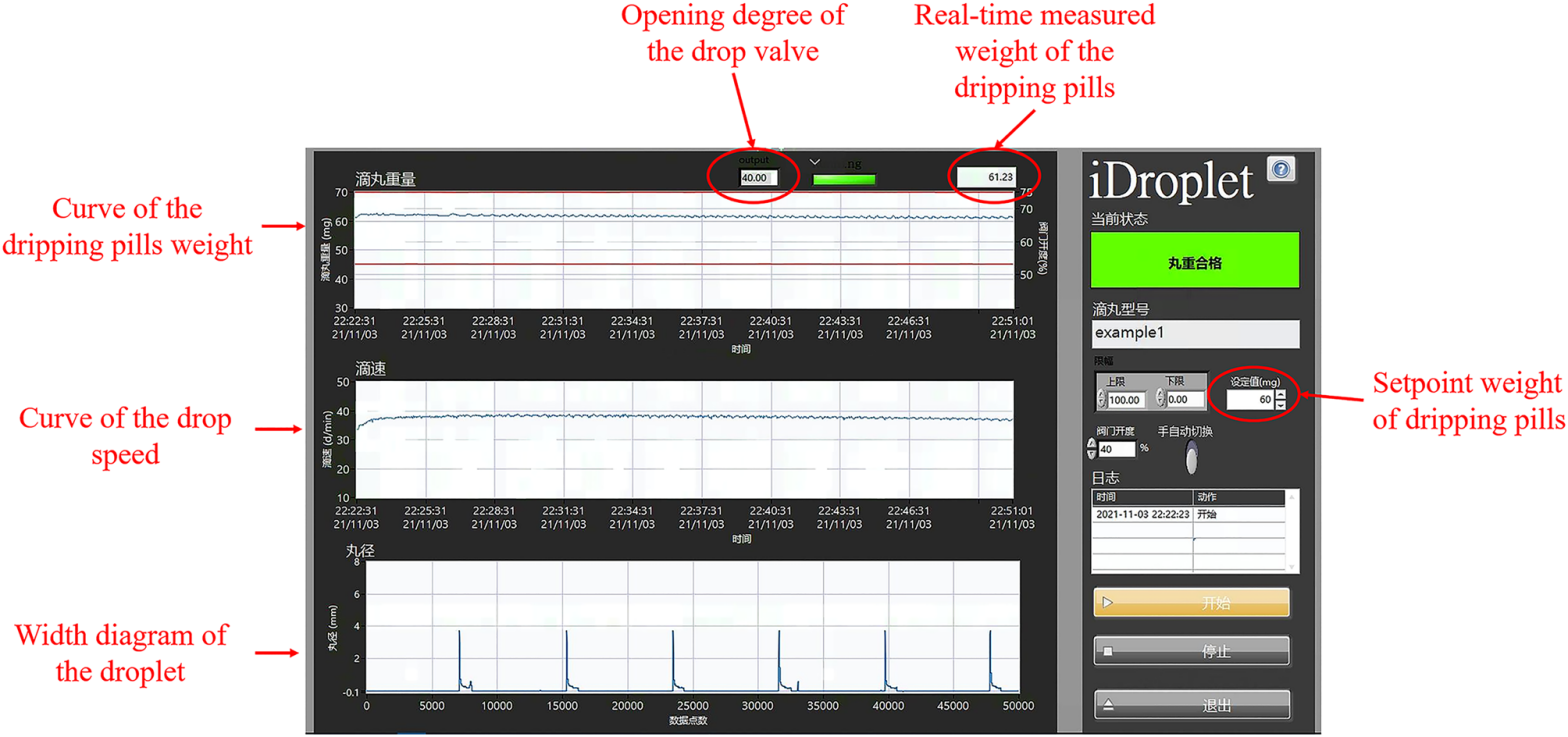
The software interface of ‘iDroplet’.

Based on the fluid dynamic theory, the dripping process comprising dispersing liquid flows out of the drop head as droplets^12,13^. The temperature of the dispersing liquid, the pressure of the liquid tank, and the opening degree of the drop valve are the critical process parameters that influence the weight of the dripping pills. The temperature of the dispersing liquid determines the fluidity and density of the liquid. The viscosity and density of the liquid decrease with increasing temperature and further affect the weight of the dripping pills. The temperature of the dispersing liquid is controlled by a heating unit installed at the out-layer of the liquid tank. The speed adjustment of the temperature is too slow; hence the temperature of the dispersing liquid is not a suitable process parameter that can be used as manipulated variable due to high hysteresis. The pressure of the liquid tank affects the pressure of vertical mobility, which determines the drop speed and the fluid system. If the pressure of the liquid tank is too high, the dispersing liquid may flow out of the drop head as a liquid jet but not as droplets. Hence, the pressure of the liquid tank is an overly sensitive process parameter, which may cause material consumption. In commercial production, the temperature of the dispersing liquid and the pressure of the liquid tank is kept constant during the whole manufacturing process. The opening degree of the drop valve affects the flow resistance of the dispersing liquid and further determines the flow pattern and the weight of the droplet. The weight of the dripping pills can be adjusted with the flexible opening degree of the drop valve. The opening degree of the drop valve was chosen as the suitable manipulated variable in this feedback control system. It was adjusted by an electric actuator (VTORK Technology VTQ50, Wuxi, China). Figure 2 shows the detailed setup used in the dripping experiments. The electric actuator was installed at the dripping device, and it controlled the opening degree of the drop valve by rotating the valve rod. The control command was ordered using a self-designed software named ‘iDroplet’ by LabView 2018 (National Instruments, Austin, TX, USA). Figure 3 is the software interface of ‘iDroplet’. Also, the software can be used to gather and save all the process data, such as real-time weight measurement of the dripping pills, opening degree of the drop valve, setpoint weight of the dripping pills, and so on.

#### Integration of closed-loop feedback control system

The ‘iDroplet’ was equipped with an in-line laser detection system and a PI controller for conducting the closed-loop feedback control strategy. A PI controller was programmed to compare the measured value with the setpoint value, calculate the error, and generate a response^14,15^. The opening degree of the drop valve (*u*(*t*)) is adapted based on the sum of the control terms, the proportional and integral term [Eq. (1)].

1 $$u(t)={u}_{0}+{K}_{c}\left(e+\frac{1}{{T}_{i}}{\int }_{0}^{t}edt\right)$$

where *K*_*c*_ is the proportional gain, *T*_*i*_ is the integral gain, *e* is the current error at time point *t*, and *u*_*0*_ is a constant controller bias.

**Figure 4:**
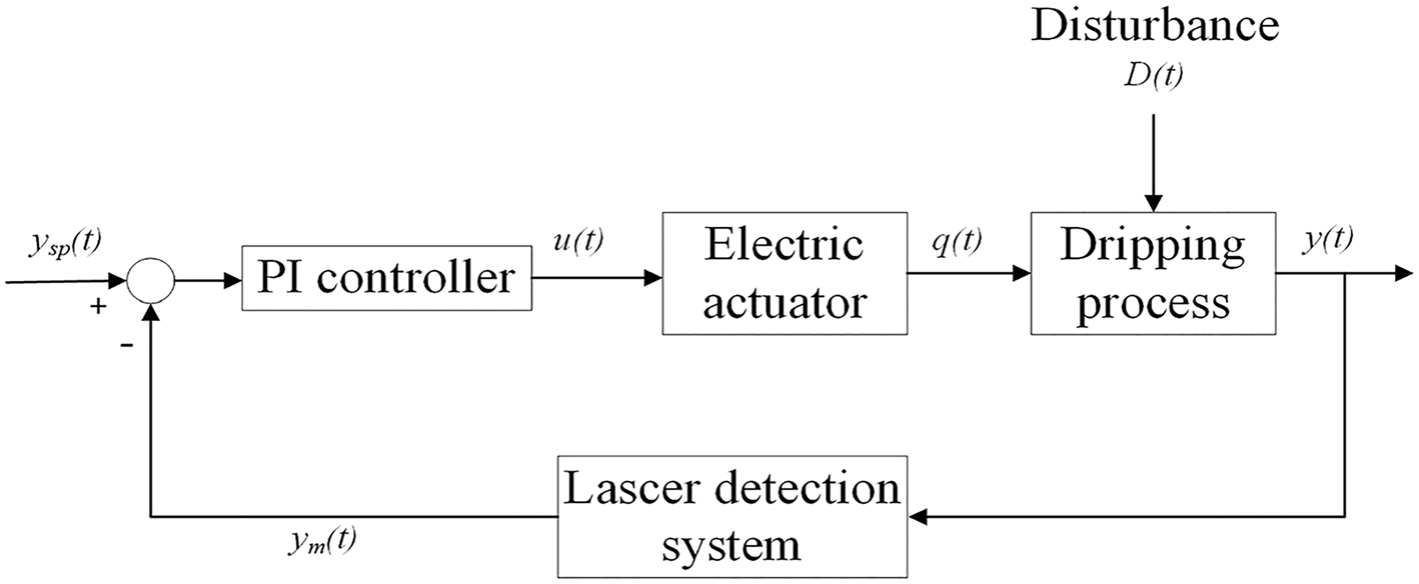
Control chart of the feedback control strategy. *q(t)* is the opening degree of the drop valve, *y(t)* is the actual value of dripping pills weight, *y*_*m*_*(t)* is the measured value of dripping pills weight, *y*_*sp*_*(t)* is the setpoint of dripping pills weight, and *D(t)* is the external disturbance.

The control chart of the feedback control strategy is illustrated in Fig. 4. The real-time measurement of the dripping pills weight was utilized as a response variable in the feedback control experiments when the opening degree of the drop valve was modified. By comparing the error between the setpoint value and the measured weight of the dripping pills, the PI controller gives a control command and acts on the electric actuator. Then the opening degree of the drop valve was adjusted by the electric actuator, and the state of the dripping process was changed. The laser detection system detects the current weight of the dripping pills and the current error between the setpoint value. The measured value was calculated and used as an index to command the next step of the electric actuator. The control system actuates the electric actuator in 8 s time intervals to minimize fluctuation levels and ensure highly effective process control.

### Evaluation of the feedback control system

Process capability index (C_pk_) was used to evaluate the efficiency of the feedback control system. The C_pk_ was calculated using Eq. (2). The target C_pk_ commonly used in the pharmaceutical industry is greater than 1.67, which corresponds to an excellent quality condition for the process^16–18^.

2 $${C}_{pk}=min\left[\frac{\mu -LSL}{3\sigma },\frac{USL-\mu }{3\sigma }\right]$$

where *LSL* is the lower specification limit, *USL* is the upper specification limit, *μ* is the specification center value, and *σ* is the standard deviation.

Different target dripping pills weights were set to determine if the control system could trace the weight back to the setpoint value. Two critical process parameters were chosen as disturbances to investigate if the control system could avoid the disturbance and trace the weight of the dripping pills back to the setpoint value. The attributes of the dispersing liquid, such as viscosity, surface tension, and density, are influenced by (which) temperature variation, which ultimately influenced the weight of the dripping pills. In commercial production, the temperature of the dispersing liquid may be different due to the low-temperature control precision of the dripping machine. Hence, we simulate several different temperatures of the dispersing liquid as the disturbance to evaluate the robustness of the feedback control system.

Furthermore, the level of the dispersing liquid in the liquid tank was decreased during the dripping process; hence the dispersing liquid needs to be supplemented continuously. The level of the dispersing liquid in the tank determines the pressure of vertical mobility and further affects the weight of the dripping pills. When the level of the dispersing liquid decreases, the weight of the dripping pills decreases simultaneously. To investigate whether the control system could reject the disturbance and trace the weight of the dripping pills back to the setpoint, the supplement of the dispersing liquid was studied as another disturbance parameter.

### Plant material collection and use permission

No permission is required for plant material as it was purchased from certifed dealer of local area.

## Results and discussion

### Tuning of the feedback control system

**Figure 5:**
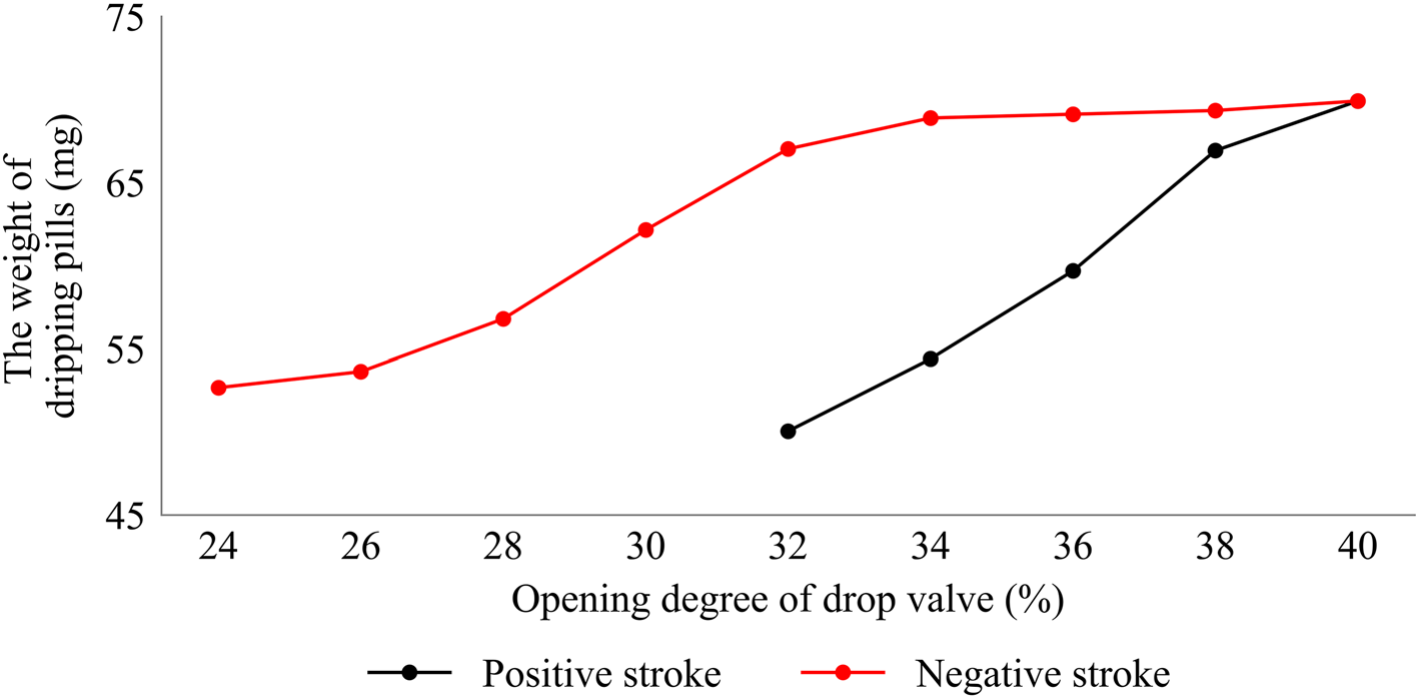
The weight of the dripping pills under positive stroke and reverse stroke.

The drop valve used in this study is a ball valve and has a different dripping pills weight response curve between the positive and reverse stroke. As shown in Fig. 5, when the drop valve is in positive stroke, the effective response range of the opening degree of the drop valve is 32–40%, while the negative stroke is 24–40%. Hence, the feedback control system must compensate for the disturbance of the attributes of the drop valve. The proportional gain should be small enough to avoid overshoot in this context.

**Figure 6:**
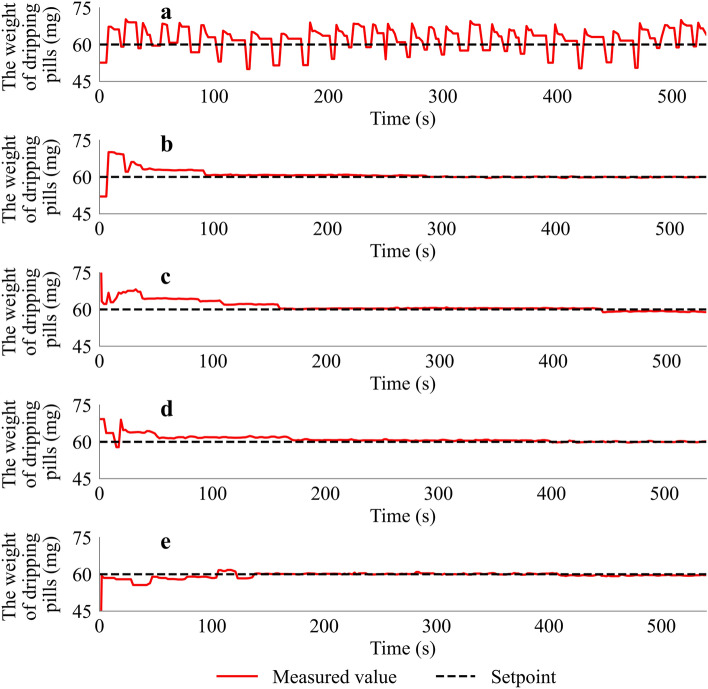
Measured weight of the dripping pills during the feedback control experiments. (**a**) *K*_*c*_ = 2.0, *T*_*i*_ = 1.0; (**b**) *K*_*c*_ = 1.8, *T*_*i*_ = 1.0; (**c**) *K*_*c*_ = 1.5, *T*_*i*_ = 1.0; (**d**) *K*_*c*_ = 1.8, *T*_*i*_ = 1.5; (**e**) *K*_*c*_ = 1.8, *T*_*i*_ = 0.5.

An optimal value for proportional and integral time should be obtained to develop an effective process control strategy. In continuation with this objective to evaluate the system oscillations, we evaluated several different values of *K*_*c*_ and *T*_*i*_, and the setpoint value was 60 mg. As shown in Fig. 6a, the results illustrate that when *K*_*c*_ is 2.0 and *T*_*i*_ is 1.0, the system oscillates with high amplitude and is hard to track the setpoint value. Then the value of *K*_*c*_ was lowered to 1.8, resulting in improved performance (Fig. 6b). The oscillations were quickly reduced with a small amplitude, and the weight of the dripping pills traced the setpoint value after 93 s. When the value of *K*_*c*_ is lowered from 2.0 to 1.8, the oscillation curve (Fig. 6c) has a significant difference. Due to the slower control process, there has been no overshoot. The measured weight of the dripping pills is adjusted and traced back to the setpoint value after 159 s.

Moreover, the values of the *T*_*i*_ are also determined. When the value of *T*_*i*_ is 1.5 and 0.5, the results indicate that similar trends of the oscillations (Fig. 6d,e) were obtained at a *T*_*i*_ value of 1.0 (Fig. 6b). However, more time must be required to reach the setpoint value (Fig. 6d), and no significant difference has been recorded (Fig. 6e). The setting (*K*_*c*_ = 1.8, *T*_*i*_ = 1) was reasonably good, and the control system can track the setpoint with a relatively high response.

### System performance of the feedback control system

**Figure 7:**
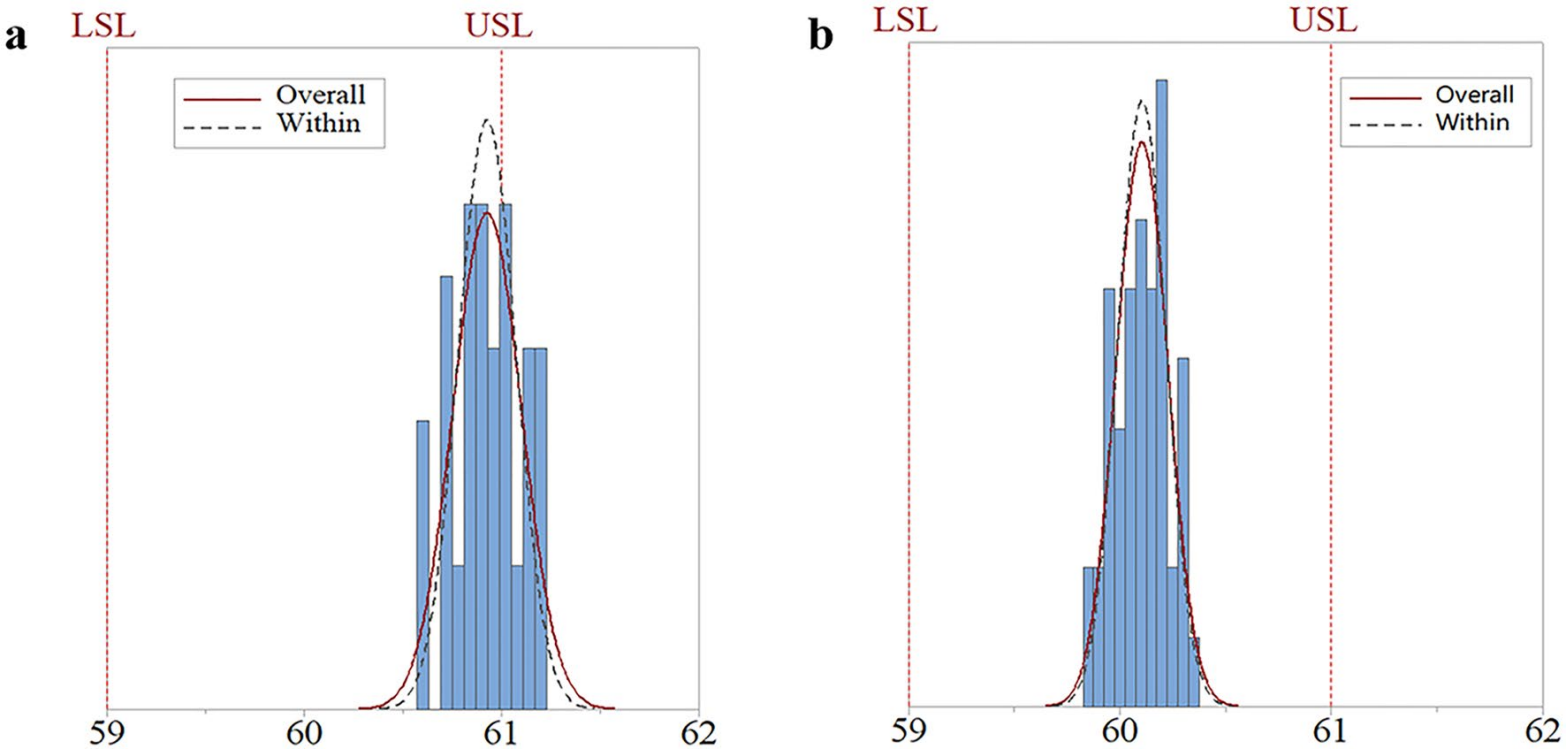
Histograms of the dripping pills weight distribution. (**a**) dripping process performed without the closed-loop feedback control system; (**b**) dripping process performed under the closed-loop feedback control system.

C_pk_ was calculated to evaluate the process capabilities of the dripping processes, which were performed without or under the closed-loop feedback control system. In this study, the USL of the dripping pills weight was 61 mg, and the LSL was 59 mg. The sample sizes were 50. As shown in Fig. 7a, the measurements were located near USL of the dripping pills weight when the dripping process was performed without the closed-loop feedback control. The C_pk_ of the dripping process, which was performed without the closed-loop feedback control, was 0.17, while the dripping process performed under the closed-loop feedback control it was 2.60 (Fig. 7b). The results indicated that the closed-loop feedback control system could efficiently improve the dripping process capability.

### Evaluation of the functionality of the feedback control system

**Figure 8:**
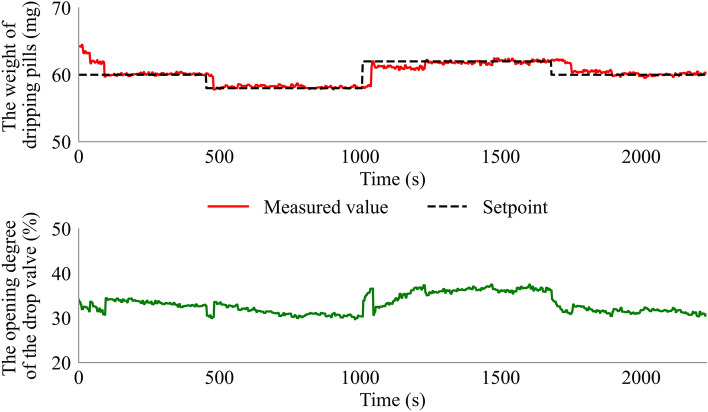
The weight of the dripping pills and the opening degree of the drop valve during the feedback control experiments.

To evaluate the functionality of the control system, three target dripping pills weights (60, 58, and 62 mg) were studied during the dripping process. The experiment conditions, including the temperature of the dispersing liquid (80 °C) and the drop distance (5 cm), were kept constant. At first, the target dripping pills weight of 60 mg was investigated. As shown in Fig. 8, a good fit between the measured value of dripping pills weight and the setpoint value was obtained after 92 s, and the fluctuation range of the dripping pills weight was 0.5 mg. Then we adjusted the target dripping pills weight to 58 mg at 454 s. The control system implemented a rapid response and adjusted the dripping pills weight to the setpoint value after 26 s, and the results were found to be within the accepted weight range of 57.81–58.58 mg. Finally, the target dripping pills weight of 62 mg was set at 1010 s, and the control system traced the dripping pills weight back to the setpoint value at 1233 s with a small amplitude of 61.43–62.23 mg. The results confirmed that the control system could respond to different target dripping pills weights. Moreover, high conformity between the weight of the target dripping pills and the measured weight of the dripping pills was obtained with a small amplitude. The control system can adjust the dripping pills weight to the setpoint value in 2 min, and the high-weight control accuracy of the feedback control system can improve the weight uniformity of dripping pills.

### Evaluation of the robustness of the feedback control system

#### Temperature of the dispersing liquid

**Figure 9:**
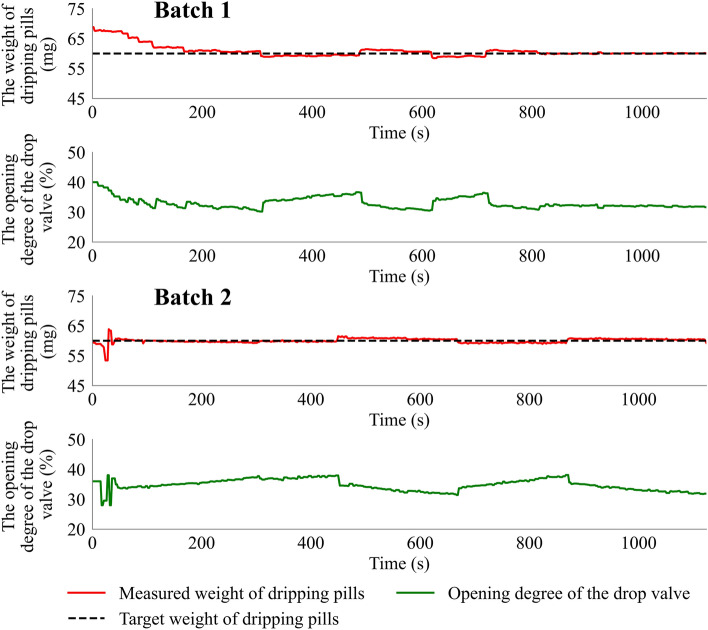
The weight of the dripping pills and the opening degree of the drop valve under different temperatures.

Two batches of the dripping process using different temperatures of the dispersing liquid were studied. The temperature of the dispersing liquid used in **Batch 1** and **Batch 2** was 70 °C and 90 °C, respectively. The target dripping pills weight was set at 60 mg. The detailed results of the measured weight of the dripping pills and opening degree of the drop valve during two feedback control experiments under different temperatures are shown in Fig. 9. In **Batch 1**, the feedback control system adjusted the measurement weight of the dripping pills rapidly and adjusted the weight to the setpoint value after 167 s, while the setpoint was traced after 41 s in **Batch 2**. The results indicated that the feedback control system provided high robustness even when the dispersing liquid was under different temperatures.

#### Supplement of the dispersing liquid

**Figure 10:**
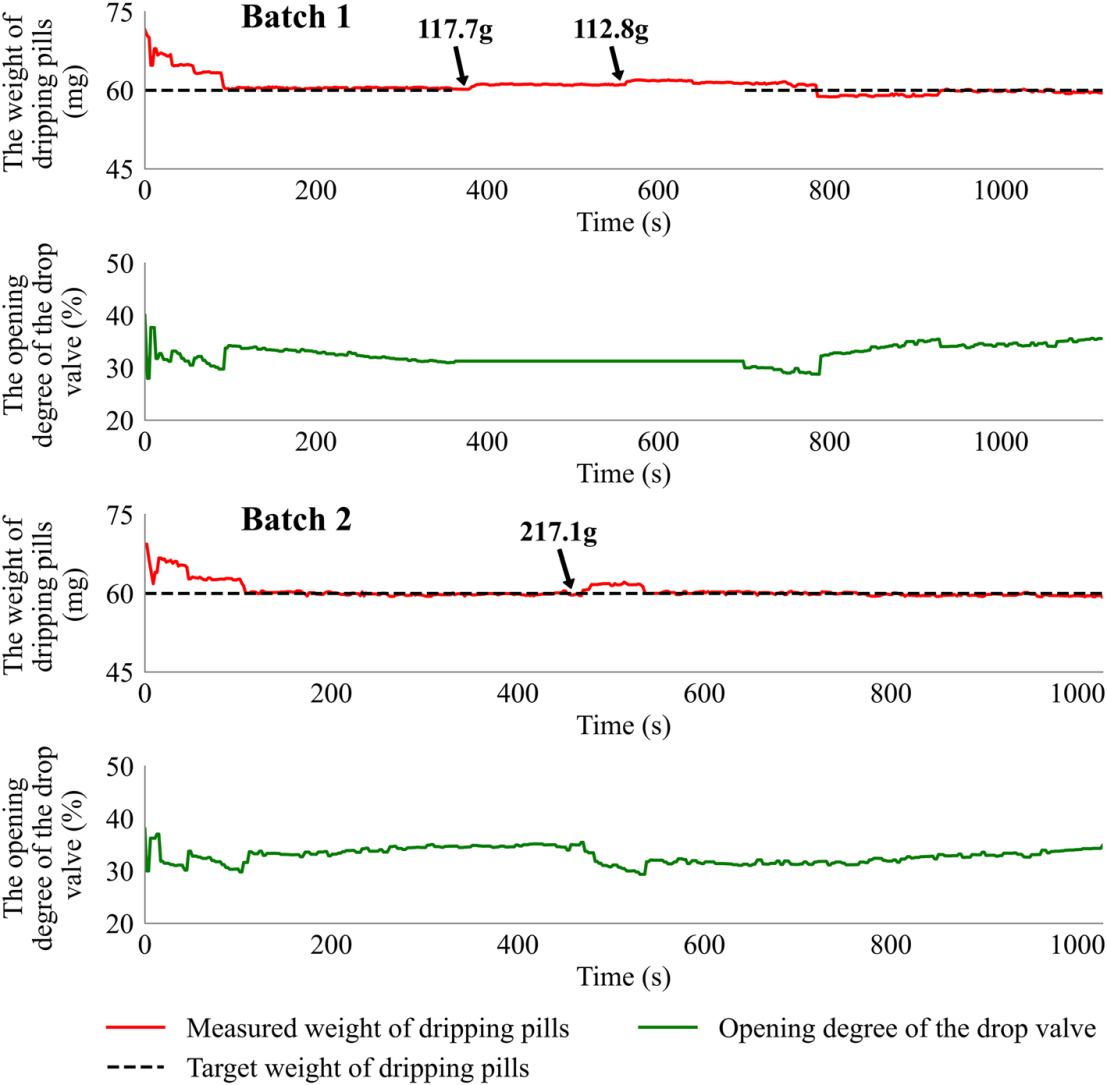
The weight of the dripping pills and the opening degree of the drop valve during two feedback control experiments. The arrow marks represent adding hot dispersing liquid to the liquid tank.

Two batches of the dripping process were studied. Two feeding processes were simulated in **Batch 1** and one in **Batch 2**. The supplementary dispersing liquid was prepared using a circulating oil bath. The target dripping pills weight was set at 60 mg. The detailed results of the dripping pills' measured weight and the drop valve's opening degree during two feedback control experiments are shown in Fig. 10. In **Batch 1**, the feedback control system adjusted the measurement weight of the dripping pills to the setpoint value after 92 s, then the weight of the dispersing liquid of 117.7 g and 112.8 g were added to the liquid tank at 382 s and 559 s, respectively. The feedback control system was turned OFF while adding the dispersing liquid. The weight of the dripping pills increased with the increasing weight of the material. The control system was turned ON at 699 s. Then the control system implemented a quick response, the opening degree of the drop valve was regulated, and the measured weight of the dripping pills was traced back to the setpoint value at 911 s. In **Batch 2**, the feedback control system was always turned ON. The weight of the dispersing liquid of 217.1 g was added to the liquid tank at 474 s. The opening degree of the drop valve was turned down, and the weight of the dripping pills was traced back to the setpoint value after 62 s with an acceptable deviation. The results indicate that the constructed feedback control system has high robustness to avoid the disturbance and trace the weight of the dripping pills back to the setpoint value.

In summary, the feedback control system can compensate the temperature fluctuation and the supplement of the dispersing liquid, implying that the feedback control system can be beneficial to ensure high product quality.

### Application of the feedback control system to *Ginkgo biloba leaf* dripping pills

**Figure 11:**
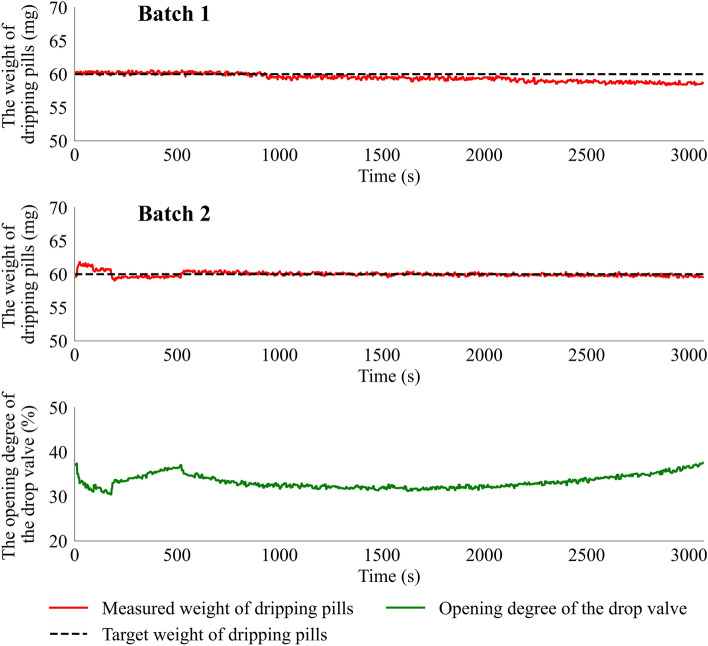
Detailed results of two batches of dripping processes.

The present research investigated two batches of *Ginkgo biloba* leaf dripping pills. One batch was performed under the feedback control system (**Batch 2**), while another was performed without the control (**Batch 1**). The experimental conditions of the two dripping processes were the same. The target weight of the dripping pills in **Batch 2** was set at 60 mg. As shown in Fig. 11, the weight of the dripping pills in **Batch 1** decreased as the dripping process prevailed. The weight range of the dripping pills was 58.31 ~ 60.58 mg, and the mean value was 59.47 mg. However, the weight of the dripping pills in **Batch 2** showed a different trend. The original weight of the dripping pills was 59.76 mg, and the feedback control system implemented a rapid response and traced it back to the setpoint value after 90 s. Then the weight of the dripping pills was kept stable with a small drift of 59.39 ~ 60.62 mg, and the mean value of the dripping pills weight was set at 59.98 mg. The results indicated that the feedback control system could effectively control the real-time weight of the dripping pills. The weight uniformity of dripping pills can be improved under the feedback control during *Ginkgo biloba* leaf dripping pills manufacturing.

## Conclusions

A real-time feedback control system was constructed to adjust the weight of the dripping pills during the dripping pill manufacturing process. A PI controller was successfully implemented using an in-line laser detection system as a PAT tool to measure the weight of the dripping pills. The opening degree of the drop valve was chosen as the manipulated variable. The results showed that the feedback control system provided excellent functionality and high robustness with an accepted deviation, and the process capability of the dripping process was improved efficiently. In summary, the constructed feedback control system could be introduced as an innovative solution to improve the quality conformity of the product and minimize the possible errors for weight measurement of the dripping pills. Moreover, it can satisfy both the availability and efficiency during the dripping pill manufacturing process. Further, the efficiency of the developed system needs to be tested in commercial plants and a wide range of raw materials for dripping pills manufacturing.

## Data Availability

The datasets generated during and/or analysed during the current study are available from the corresponding author on reasonable request.
